# Stability of BSE infectivity towards heat treatment even after proteolytic removal of prion protein

**DOI:** 10.1186/s13567-021-00928-8

**Published:** 2021-04-16

**Authors:** Jan P. M. Langeveld, Anne Balkema-Buschmann, Dieter Becher, Achim Thomzig, Romolo Nonno, Olivier Andréoletti, Aart Davidse, Michele A. Di Bari, Laura Pirisinu, Umberto Agrimi, Martin H. Groschup, Michael Beekes, Jason Shih

**Affiliations:** 1 Department of Infection Biology, Wageningen Bioveterinary Research (WBVR), 8221RA 39 Lelystad, The Netherlands; 2 Institute of Novel and Emerging Infectious Diseases, Friedrich-Loeffler-Institut, 17493 Greifswald-Insel Riems, Germany; 3 MICROMUN, Institut Für Mikrobiologische Forschung GmbH, 17489 Greifswald, Germany; 4 Prion and Prionoid Research Unit, Robert Koch-Institute, 13353 Berlin, Germany; 5 Department of Food Safety, Nutrition and Veterinary Public Health, Istituto Superiore Di Sanità, 00161 Rome, Italy; 6 UMR INRAE/ENVT 1225 IHAP, École Nationale Vétérinaire de Toulouse, 31300 Toulouse, France; 7 Department of Poultry Science, North Carolina State University, Raleigh, NC 27695-7608 USA

**Keywords:** Prion, PrP, Molecular mechanism, BSE, Zoonotic, Infectivity, Strain, Heat, Inactivation, Bioassay

## Abstract

The unconventional infectious agents of transmissible spongiform encephalopathies (TSEs) are prions. Their infectivity co-appears with PrP^Sc^, aberrant depositions of the host’s cellular prion protein (PrP^C^). Successive heat treatment in the presence of detergent and proteolysis by a keratinase from *Bacillus licheniformis* PWD-1 was shown before to destroy PrP^Sc^ from bovine TSE (BSE) and sheep scrapie diseased brain, however data regarding expected reduction of infectivity were still lacking. Therefore, transgenic Tgbov XV mice which are highly BSE susceptible were used to quantify infectivity before and after the bovine brain treatment procedure. Also four immunochemical analyses were applied to compare the levels of PrP^Sc^. After heating at 115 °C with or without subsequent proteolysis, the original BSE infectivity of 10^6.2–6.4^ ID_50_ g^−1^ was reduced to a remaining infectivity of 10^4.6–5.7^ ID_50_ g^−1^ while strain characteristics were unaltered, even after precipitation with methanol. Surprisingly, PrP^Sc^ depletion was 5–800 times higher than the loss of infectivity. Similar treatment was applied on other prion strains, which were CWD1 in bank voles, 263 K scrapie in hamsters and sheep PG127 scrapie in tg338 ovinized mice. In these strains however, infectivity was already destroyed by heat only. These findings show the unusual heat resistance of BSE and support a role for an additional factor in prion formation as suggested elsewhere when producing prions from PrP^C^. Leftover material in the remaining PrP^Sc^ depleted BSE preparation offers a unique substrate for searching additional elements for prion infectivity and improving our concept about the nature of prions.

**Supplementary Information:**

The online version contains supplementary material available at 10.1186/s13567-021-00928-8.

## Introduction

Prions are infectious agents of transmissible spongiform encephalopathies (TSEs) or prion diseases [1]. The infectivity is dependent on a conformationally malformed state (PrP^Sc^) of the physiological protein PrP^C^, a cellular membrane protein with an as yet unclear function. The mechanism of transformation of this host encoded PrP^C^ to PrP^Sc^ includes refolding and aggregation. PrP^Sc^ is partially resistant to digestion with proteolytic enzymes, usually proteinase K (PK). During proteolysis—often in the presence of detergent—the PrP^Sc^ molecules become N-terminally truncated while the remaining C-terminal part (PrP^res^) after dissociation and unfolding is characterized by a triplet of a diglycosylated, monoglycosylated and non-glycosylated PrP fragment in the 18–30 kDa molecular mass range.

The proof that PrP^Sc^ represents infectivity was first based on biomathematical and extensive biochemical work with hamster scrapie [1–5]. Definitive proof that the presence of PrP^C^ is a prerequisite for TSE infection was presented from PrP-less mice, goats and cells, and by the production of infectivity from recombinantly expressed and purified PrP [6–14]. Another argument for the validity of the role of PrP in the agent is the close relation between susceptibility/resistance in e.g. sheep, goats and humans and genetic polymorphisms in the *PRNP* coding region [15–17]. Yet, the very reproducible strain properties characteristic for TSEs are not yet explained sofar. These might be dependent on the presence of lipid, polyanionic glycans or nucleic acid fragments in the agent or during PrP^Sc^ formation [9, 13, 18–20]. From observations with bovine spongiform encephalopathy (BSE) inoculated in in-bred wildtype mice it was even postulated that an additional unidentified agent may be essential for transmission while PrP^Sc^ would be involved in species adaptation [21].^1^

Previously, we found that *B. licheniformis* PWD1 keratinase (KE) at 50 °C could reduce PrP^Sc^ by more than 99.9% after autoclaving for 40 min at 115 °C in the presence of the detergent sarkosyl at neutral pH [22]. The material used in these experiments was brain stem from cattle and sheep clinically affected respectively by BSE and scrapie. Other investigators found proteases which already had a substantial PrP^Sc^ degrading effect even without heating above 100 °C or the presence of detergents in the homogenate while pH varied between 7–12 and temperatures between 37–70 °C [23–25]. According to these results, there is no direct correlation between PrP^Sc^ level and infectivity. This weakens the prion hypothesis which in part is based on a positive correlation between the two parameters [5, 26]. Further confusing are examples of infectivity related to protease sensitive PrP^Sc^ [27–29].

In this study we investigated whether our PrP^Sc^ removal from BSE infected cow brain using heating at 115 °C and enzymatic proteolysis goes together with removal of infectivity in the highly sensitive transgenic Tgbov XV mice expressing bovine PrP. The presence of PrP^Sc^ was tested in Western blotting and several biochemical methods. We also compared the effect of this brain treating methodology when applied on three other prion isolates with short incubation times respectively 263 K scrapie strain in hamsters, chronic wasting disease (CWD) strain CWD1 in bank voles and sheep PG127 scrapie in tg338 mice that are expressing sheep PrP_VRQ_.

## Materials and methods

### Antibodies

PrP-specific monoclonal antibodies (mAb’s) used were: SAF34, Bar224, 12B2, 9A2, 3F4, 6C2, 12F10, L42, 6H4, Sha31, SAF84, 94B4, F99/97.6.1 [30–37]. Their linear specificities on PrP have been described and further confirmed by Pepscan analysis [38] as follows (bovine PrP numbering, 6 octarepeats): 62QPHGGGW92 (SAF34), 101WGQGG105 (12B2), 110WNK112 (9A2), 117KTNMKHV113 (3F4), 122HVAGAAA128 (6C2), 152FGSDYEDRYYR162 (Bar224), 154 NDYEDRYYRE163 (12F10), 156YEDRYY161 (L42), 156YEDRYYRE163 (Sha31), 156YEDRYYREN164 (6H4), 174YRPVDQY180 (SAF84), 198HTVTTTTK205 (94B4) and 229YQRE232 (F99/97.6.1).

### Proteolytic enzymes

Lyophilized keratinase (KE) was used in purified form (1.4 × 10^4^ azocaseine-U mg^−1^) [39, 40]. Proteinase K (PK) was purchased as lyophilized product (Merck 1.24568; 30 mAnson-U mg^−1^).

### Tissues

Bovine BSE brains were from the rostral part of the obex of a British clinically and histologically confirmed positive BSE cow (UK case 97/0913, kind gift from APHA Weybridge at UK), and obex tissues from confirmed BSE positive Dutch cases NL6 (clinically positive), NL11 and NL19 (clinically healthy at slaughter) as well as from BSE confirmed negative cattle. Hamster brains infected with the 263 K scrapie strain were supplied by RKI Berlin, sheep PG127 scrapie brain isolate was second oral passage material in VRQ/VRQ sheep prepared at ENVT Toulouse, CWD1 isolate was passaged three times in bank voles with PrP genotype 109I/I (Bv109I).

### Ethical statement

Animal experimentations were performed in 2004–2009 according to the prevailing regulations of European directives (86/609/EEC) as well as in compliance with the respective national and institutional legislations. The number of animals used were kept at the lowest as considered necessary for the experiments in line with the three R’s concept: replace, reduce and refine. This means per dose group 10–16 animals for Tgbov XV mice, and six for the other rodent bioassays.

### Preparation of inocula

Brain materials from cattle and rodents were subjected to similar procedures with disposable equipment. Homogenizations were carried for 45 s at 23 000 rpm in Prypcon vials with a MediFASTH apparatus (Consul AR SA; Villeneuve, Switzerland). Negative tissues were first prepared before the positive ones, and dilutions of samples were performed with changing pipet tips for every next dilution step.

Brain stem material from cattle and sheep and rodent brains were homogenized as 10% (w/t) tissue samples either in physiological saline (PS) or in the presence of 2% sarkosyl as detergent in 50 mM sodium phosphate pH7.5.

The detergent containing homogenate was aliquoted. One aliquot was not heated. The other aliquot was placed in a 28 mL Bijou bottle covered with a paper fiber stopper and autoclaved in a pressure cooker at 115 °C for 40 min as described [22]. One aliquot of the heated material was left undigested, and another part was further subjected to digestion for 60 min at 50 °C with 25–50 azocaseine-U KE mg^−1^ tissue equivalents (TE) unless otherwise stated. For the scrapie and CWD1 experiments an additional heated aliquot was digested with 0.015–0.005 mAnson-U PK mg^−1^ TE.

Undigested and digested homogenates were further diluted to 1% original tissue concentration in PS or, where mentioned, first precipitated with nine volumes of cold methanol by centrifugation for 10 min at 16 000 × *g* and resuspended in PS. After centrifugation of digested and heated material with or without methanol, a pellet was visible only when methanol was used. The 1% tissue homogenates were heated for 20 min at 80 °C and stored at −80 °C till use.

### Animal studies

Tgbov XV mice overexpressing bovine PrP that are highly sensitive to bovine BSE infection were used for challenges by intracerebral inoculation with 20 μL of 1% (w/t) and lower doses tissue homogenate [41]. Inocula were prepared (see paragraph below) from the British BSE case. End-point titers expressed as ^10^log infectious doses per g tissue (ID_50_ g^−1^) were determined applying the Spearman-Kaerber method [42].

Syrian hamsters, bank voles (Bv109I) and transgenic mice expressing the sheep PrP_VRQ_ (Tg338) were used respectively for studies with 263 K scrapie infected hamster, CWD1_109I_ from Bv109I after 3^rd^ passage and sheep scrapie isolate PG127. Per animal 20 μL (50 μL in case of hamsters) of 1% (w/t) and lower doses of brain homogenate were intracerebrally inoculated as described [43–45]. Animals were culled when positive for clinical signs such as tremor of head or whole body, incoordination of gait, difficulty in rising from a supine position, and impairment in their capacity to feed. Animal brains were postmortem also checked for the presence of PrP^res^ in Western blotting and in case of Tgbov XV mice also first by PrP^res^ with TeSeE® SAP combination kit (Bio-Rad).

Infectivity titers were based on PrP^Sc^ detection in Tgbov XV mice and in the other three rodent lines on survival times.

### Biochemical analyses

Bovine brain homogenates were tested for presence of PrP using Western blotting as previously described [22]. Running buffer was either 3-N-morpholino)propane sulfonic acid (MOPS) or 2-(N-morpholino) ethane sulfonic acid (MES). Staining of protein in gels was performed with the SilverXpress™ kit (Thermo Fisher Scientific) followed by destaining of silver and restaining with Coomassie brilliant blue [46, 47].

Brain material of Tgbov XV mice was tested for PrP^res^ with the TeSeE® purification and detection kit (Biorad). Borderline and negative cases in TgBov XV mice were checked by Western blotting after a concentration procedure involving pelleting by ultracentrifugation at 540 000 × *g* [41]. PrP^res^ detection by Western blotting in hamsters with mAb 3F4, bank voles with mAb SAF84 and tg338-mice brain with mAb Sha31 followed described methods [43, 45, 48].

For further establishing presence of PrP^Sc^ or PrP^res^ in BSE related samples, three different EC approved commercial enzyme-linked immunosorbent assays (ELISAs) for BSE testing were carried out: TeSeE® SAP combination kit (Bio-Rad), HerdCheck BSE Ag test (IDEXX Europe BV) and CediTect® BSE test (Prionics Lelystad BV). The general principle of these tests is described in Additional file 1.

Reference internal control samples were exactly treated as in routine testing is required. However, for each of the three tests, study samples were first precipitated with nine volumes of cold methanol, centrifuged at 16 000 × *g* for 10 min. The pellets containing 5.45 mg TE per vial were kept frozen until use. On day of analysis, pellets were taken up in the kit specific solution before including in the tests.

Pellets for the TeSeE kit were resuspended in denaturing kit buffer C and were heated as in the test kit for denaturation and subjected to the analysis in ELISA. Per well 3 mg TE were tested.

In the HerdCheck test, the pellets were resuspended in 600 μL test kit homogenization buffer and incubated at ambient temperature for 10 min. Then, 120 μL was mixed with 30 μL of plate diluent of which 100 μL was added per well of 96 wells IDEXX plate that contains Seprion ligand for binding PrP^Sc^ aggregates. Further denaturation to open bound aggregates for antibody binding was performed according to the kit protocol. Per well 0.7 mg TE were tested.

In the CediTect BSE test, pellets of the study samples were resuspended in 100 μL kit lysis buffer and after resuspension further ten times diluted with kit lysis buffer of which 50 μL (0.5 mg TE) was applied per well in each of two PVDF filter plates. Further procedure was according to the test protocol which means that after washing with phosphate buffered saline (PBS) by filtration, one plate was treated with PBS and the other with 5 M guanidinium thiocyanate in PBS and the other with PBS only. Per well 0.5 mg TE were tested.

## Results

### PrP^Sc^ digestion in heat treated cattle brain

**Figure 1:**
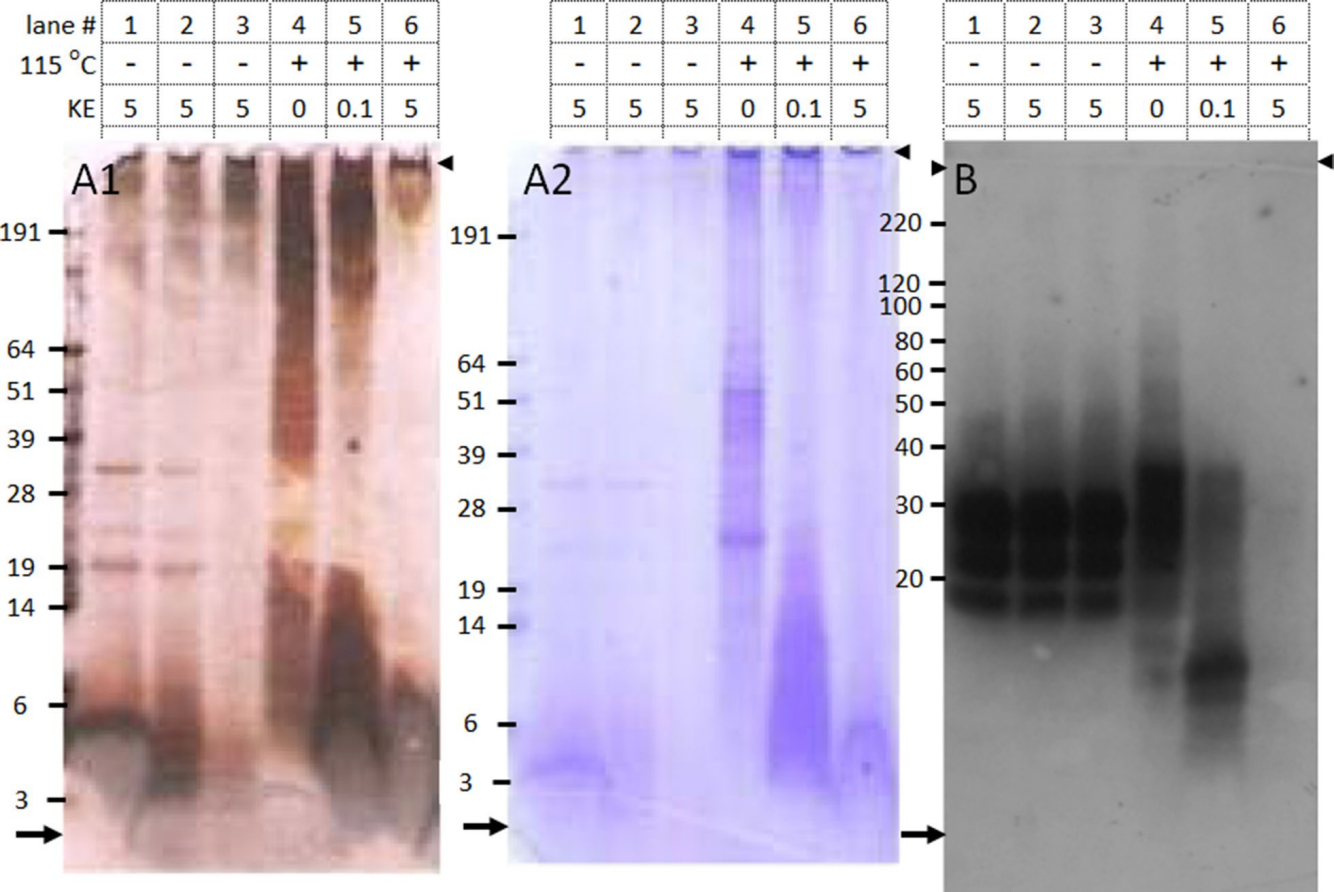
**Digestion of total protein in BSE infected bovine brain homogenate.** Panels A and B were derived from one SDS-PAGE gel, cut into two parts. Similar samples were loaded in the two parts before staining. Staining: A1, total protein with silver; A2, total protein with Coomassie brilliant blue after destaining the silver from A1; B, PrP staining after Western blotting using a mixture of antibodies 9A2 and 94B4 (each at 0.2 μg mL^−1^). Heating in detergent containing homogenization buffer is indicated in the “115 °C” row. KE row shows the azocaseine-U mg^−1^ tissue equivalents. Tissue equivalents applied: 250 μg per lane except for lane 4, only 62 μg to prevent overstaining. Samples in lanes 1 and 4–6 not precipitated, in lanes 2 and 3 precipitated respectively with methanol and 1-propanol. Molecular mass markers used were SeeBlue in A and B, and MagicMark XP in C, for which migration positions are indicated in kDa at the left. Gel was run in MES buffer. Top and running front are indicated with arrow heads and arrows, respectively.

Heating alone in the presence of 2% sarkosyl at 115 °C of homogenates prepared from brain tissue of a British confirmed BSE cow with clinical signs did not lead to significant loss of PrP^Sc^ immunoreactivity (Figure 1B, cfr. lane 4 with lanes 1–3) as was previously also shown [22]. Subsequent digestion with keratinase (KE) at increasing enzyme concentrations showed that all PrP material already disappeared at 5 KE-units per mg tissue equivalents (TE) (Figure 1B, lane 6). Staining with silver and Coomassie brilliant blue both showed that at this low enzyme/tissue ratio proteins were degraded to peptides migrating at 6 kDa and lower and to proteins with molecular masses of 300 kDa and higher (Figure 1 panels A1-2, lanes 6). However only in heated non-digested sample the ≥ 300 kDa fraction was reactive with PrP specific antibodies, but not after digestion with KE (Additional file 2 cfr. lane 4 with lane 7). This means that this large size protein material is accessible for PrP-specific antibodies. Precipitation with methanol and 1-propanol did show that the former treatment yielded acceptable recoveries of both PrP material and other proteins (Figure 1, panel A1, cfr. lane 1 with lanes 2 and 3). The level of PrP-reactive material was roughly the same between non-heated digested (lane 1) and heated non-digested or slightly digested homogenate (lanes 4 and 5).

**Figure 2:**
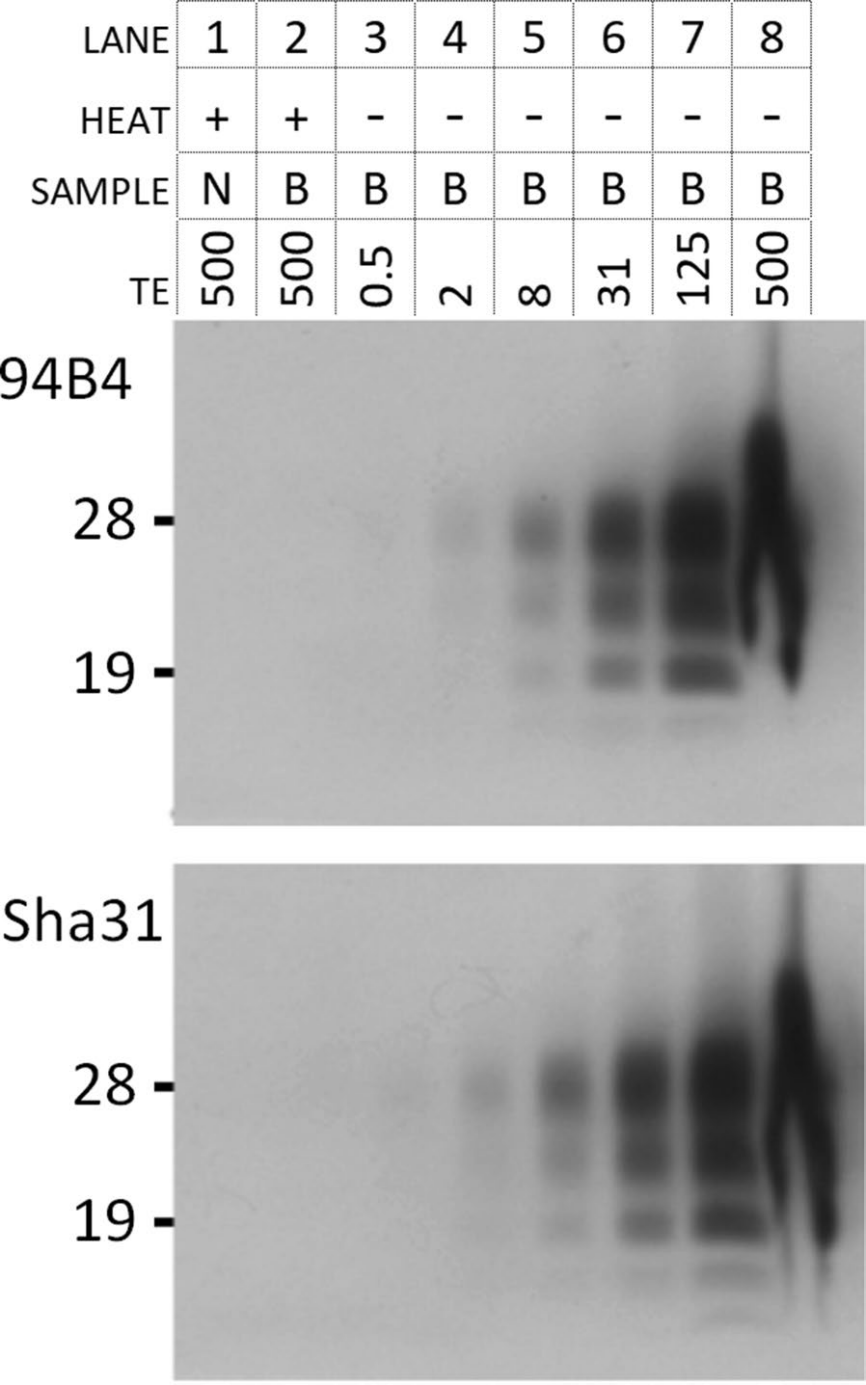
**Dilution series of keratinase digested non-heated BSE material to quantify the PrP**^**Sc**^** removal by keratinase in the heated sample.** Lanes 1 and 2 represent heated and digested BSE-negative (N) and positive (B) material, while lanes 3–8 show a non-heated and digested BSE-sample in a four-fold dilution series in reverse order. Amount of tisssue equivalents (TE) applied per lane are indicated in µg. The signal of PrP^Sc^ in lane 2 was similarly negative to that of the BSE-negative sample in lane 1, and lower than the lowest amount applied of heated and non-digested BSE sample in lane 3 i.c. less than that in the 0.5 µg TE lane. This means that removal of PrP^Sc^ from 500 µg TE in lane 2 was more than 99.9% based on the applied TE which corresponds to a > 1000 × reduction factor. The parallel blots were stained with antibodies 94B4 and Sha31 (0.1 µg mL^−1^). TE in µg, gels were run in parallel in MOPS buffer.

In heated and KE-digested brain material, the extent of PrP^Sc^ removal by keratinase from high-titer central nervous system tissue of the BSE infected cow was above 99.9% (> factor 1000x) by Western blotting with mAb’s 98B4 and Sha31 (Figure 2). Other mAb’s yielded the same outcome such as SAF34, 9A2, 12B2, 6C2, 12F10, L42, SAF84 and F99/97.6.1. This infers that the destruction of PrP had involved the whole molecule.

Three different ELISA tests were used for quantifying the presence of PrP^Sc^, each using a different property for immobilisation before further analysis, all using PrP specific mAb’s and each including a denaturation treatment to enable access of antibodies. These different properties were: 1 in TeSeE test the capture of PrP^res^ by PrP specific antibody coated to polystyrene, 2 in the IDEXX HerdCheck test the binding of PrP^Sc^-fibrils by Seprion ligand immobilized to polystyrene, and 3 in the Cedi-Tect BSE test the level of unfolding of PrP^res^—adsorbed to PVDF filters—which reflects its aggregated condition. In these tests PrP^Sc^ removal by keratinase in heated samples reached values 99.7–99.96% or alternatively PrP^Sc/^PrP^res^ reduction factors of > 2500x, > 333x, > 500x, and > 2500 × in respective TeSeE, HerdCheck, and Cedi with 9A2 and 94B4. In fact, each outcome was within borderline background (Table 1).

**Table 1:** Testing for presence of PrP^**Sc**^** in BSE samples subsequently treated with heat and digestion by keratinase**

1, test (principle)	2, antibody	3, measuring unit	4, test-control	5, non-heated / digested	6, heated / digested	7, PrP^Sc^ signal as % of non-heated digested (reduction factor)^c^
TeSeE (capture)^a^	SAF32, Bar224	OD	2.288^b^	0.020 ± 0.016	0.009 ± 0.003 [0.012]	< < 0.04% (> 2500)
					(0.009 ± 0.004) [0.013]	
HerdCheck (Seprion ligand binding)	?	OD	3.73	1.93 ± 1.13 [3.06^b^]	0.07 ± 0.04 [0.11]	< < 0.3% (> 333)
					(0.06 ± 0.06) [0.12]	
CediTect BSE (conformation dependent)	9A2	d/n	170	131.8 ± 48.5 [180.3^b^]	1.5 ± 0.5 [2.0]	< 0.2% (> 500)
					(1.6 ± 0.1) [1.7]	
	94B4	d/n	255	141.8 ± 91.4 [233.2^b^]	2.1 ± 1.3 [3.4]	< 0.04% (> 2500)
					(2.0 ± 1.4) [3.4]	

Values in tests 1—3 are presented as averages ± SD of four BSE positive cases. Between parentheses BSE negative cases. Between brackets sum of average plus maximum SD values.

Absence of PrP^Sc^ in heated and protease digested bovine brain homogenates. Differently treated homogenates from confirmed BSE positive cattle (*n* = 4, two clinical cases, two cases found at healthy slaughter) were subjected to three different immunochemical tests. Also four similarly treated homogenates from confirmed BSE negative brains were included. Values in columns 5 and 6 are presented as averages ± SD of four cases; between parentheses values found in the confirmed BSE negative cases. Between brackets sum of average value plus maximum SD value. Only the IDEXX and CediTect tests appeared able to recognize PrP^Sc^ in non-heated and KE-digested samples. The BSE positive reference controls—column 4—in the TeSeE and CediTect tests have been digested with PK exactly according to the manufacturer’s instructions and were performed on regular BSE positive samples from routine testing. The IDEXX test recognizes intact PrP^Sc^ as well as PrP^res^. The CediTect assay was applied with antibodies 9A2 and 94B4 which are specific respectively for the N-terminal and C-terminal region of PrP^res^. Tissue amounts per tested well were for TeSeE, IDEXX and CediTect test respectively 3, 0.6 and 0.5 mg wet tissue weight per well.

^a^The Biorad TeSeE ELISA does not recognize PrP^res^ in the non-heated KE digests (column 5) since the PrP octarepeat needed for recognition by the capture antibody SAF34 is fully removed during digestion with the keratinase where the conditions differ from those used for the test control. Therefore, for this ELISA the value of the positive test control in column 4 was used for calculation of the percentage residual PrP^Sc^ in column 7 since the digested non-heated cases do not bind to the catching antibody where the correct conditions for retaining the SAF34 epitope could not be applied in our digestion system with keratinase.

^b^The 100% PrP^Sc^ values of the samples in the different tests before heat treatment were used for calculating the fraction of remaining PrP^Sc^ signals of column 7.

^c^Calculations for column 7: value of BSE positives (column 6, average value plus maximum SD) – value of BSE negatives (column 6 between parenteses, average value plus maximum SD) / 100% PrP^Sc^-values mentioned in footnote ^b^.

Taken together, from these three different biochemical analyses using a range of different PrP specific antibodies, it was not possible to show the presence of any left-over PrP^Sc^ in the heat-treated, keratinase digested, bovine brain.

### Infectivity of BSE material in Tgbov XV mice

**Figure 3:**
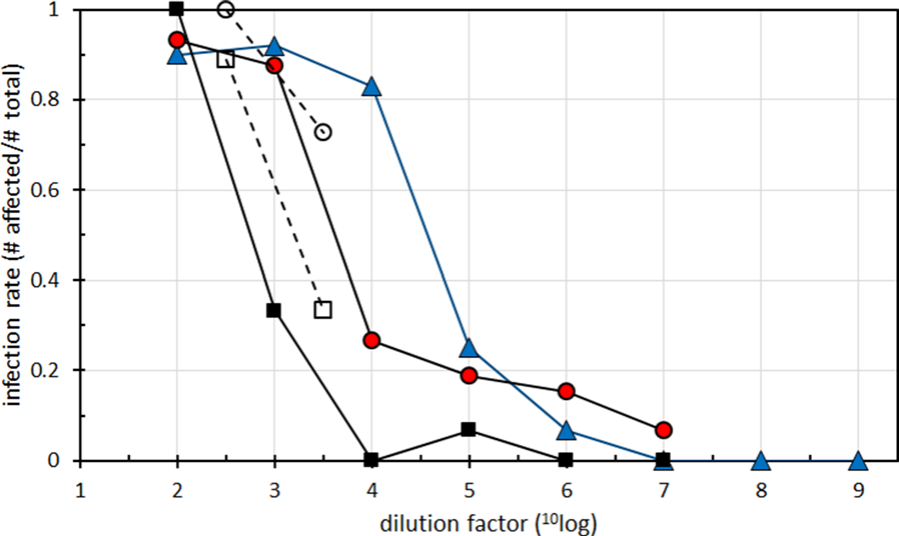
**Infectivity of heat-treated BSE homogenate remains after PrP**^**Sc**^** removal with keratinase.** Infectivity diagnosis was based on PrP^Sc^ tests in Tgbov XV mice. Symbols: squares and circles represent respectively non-digested and KE-digested material, triangles the end point titration data of homogenate without detergent/heat pretreatment. Solid lines, data from 1^st^ experiment; broken lines data from 2^nd^ experiment. The data can be found in the Additional file 3.

Infectivity of untreated cow brain homogenate used in the challenge experiments was estimated at 10^6.2^–10^6.4^ ID_50_ g^−1^, a value usual for BSE samples in mice transgenic for bovine PrP (Tgbov XV) [41, 49]. Heating at 115 °C led to an infectivity titer change down to 10^4.6^ corresponding to a 40–60 fold titre loss (Table 2). A precipitation step with methanol applied on non-digested samples did increase titers 2- and fivefold for respectively non-heated and heated material which indicated that the precipitation of BSE agent was very effective. Surprisingly, subsequent proteolytic removal of PrP^Sc^ did not further remove infectivity, but it rather led to a 12.5-fold increase of infectivity (from 10^4.6^ to 10^5.7^). For confirmation of these results, we repeated these measurements with newly generated inoculum with dilutions around the critical doses 10^2.5^ and 10^3.5^. Results agreed with those in the previous experiment: digestion of heat-treated brain homogenate did not additionally remove infectivity but rather increased the infectivity (Figure 3). Thus, here a situation is encountered where removal of all detectable forms of PrP from prions did not further reduce infectivity (Additional file 3).

**Table 2:** Infectivity measured by PrPSc positivity rates in Tgbov XV mice inoculated with brain homogenates after different treatments

Dose	No detergent	Detergent		Detergent; heat		Detergent; heat; keratinase	
(^10^log g mL^−1^)	no ppt	no ppt	ppt	no ppt	ppt	no ppt	ppt
−2	9/10 (2)	n.d	n.d	15/15 (0)	12/13 (2)	14/15 (0)	15/15 (0)
−3	12/13 (0)	n.d	n.d	5/15 (0)	14/15 (0)	14/16 (0)	n.d
−4	10/12 (0)	n.d	n.d	0/15 (0)	2/13 (2)	4/15 (1)	n.d
−5	3/12 (0)	3/15 (0)	10/15 (0)	1/15 (0)	n.d	3/16 (0)	n.d
−6	1/15 (0)	3/13 (2)	1/14 (1)	0/15 (0)	n.d	2/13 (2)	n.d
−7	0/15 (0)	2/15 (0)	1/14 (1)	0/15 (0)	n.d	1/15 (0)	n.d
−8	0/15 (0)	0/14 (1)	1/15 (0)				
−9	0/14 (1)	0/15 (0)	0/15 (0)				
^10^log ID_50_ g^−1^	6.2	6.4**	6.7**	4.6	5.3*	5.7	

Disease rates were estimated as number of animals confirmed positive (based on testing for PrP^Sc^) and the total number of animals that lived longer than 100 days post inoculation. Between parentheses the number of animals per dose group not included for disease validation because of early death i.e. < 100 days post inoculation. These animals were PrP^Sc^ negative by Western blotting. Dose was calculated using the percentages per group in the formula of Spearman and Kaerber. N.d.: not done; ppt = precipitated with methanol. Asterisks * and **: for enabling calculations, figures for the n.d. disease rates were borrowed per equal dose group respectively from the columns detergent/heat/no ppt and no detergent/no ppt.

**Figure 4:**
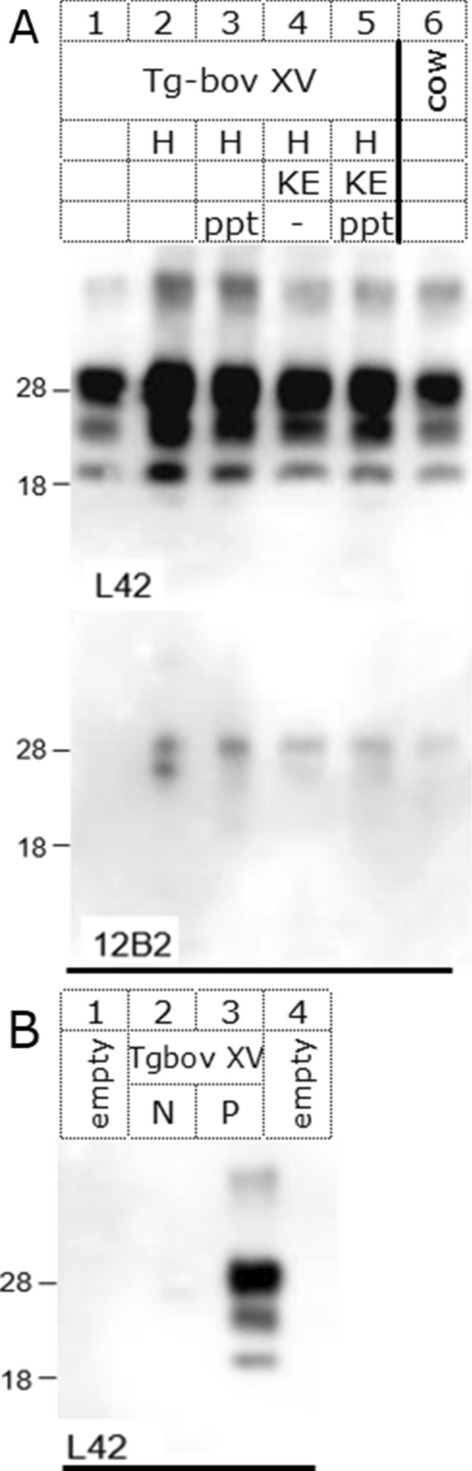
**PrP**^**res**^** patterns in diseased Tgbov XV mice brain inoculated with differently treated bovine BSE materials.** All samples digested with PK. Panel A: two similar blots developed with different antibodies as indicated. Lane 1–5, inoculated Tg bovXV mice samples; lane 6, BSE positive bovine brain. Inocula used: lane 1, BSE in physiological saline; lanes 2–5, heated BSE homogenate of which lanes 2–3 not digested with KE and lanes 4–5 digested with KE; lanes 2 and 4, inocula precipitated in methanol. Panel B: blot of control material from Tgbov XV mice experimentally infected with either brain from a clinically affected and BSE confirmed cow (lane 3) or from a confirmed BSE negative cow (lane 2). Lanes 1 and 4, empty. Migration position of molecular mass markers are indicated at the left plus their size in kDa. Antibody concentration used for L42 and 12B2 was respectively 0.5 and 0.2 µg mL^−1^. Antibodies 9A2 and 94B4 yielded similar results as L42 (not shown).

Moreover, in mice infected with these PrP depleted samples the triple band pattern of PrP^res^ yielded a typical classical BSE strain profile with respect to migration position (e.g. lower band migrating at 19 kDa), a minimal 12B2 reactivity and the diglycosylated fraction (upper band) as the major PrP band similar to the sample used for challenge (Figure 4).

### Infectivity of other TSE materials with short incubation times

**Figure 5:**
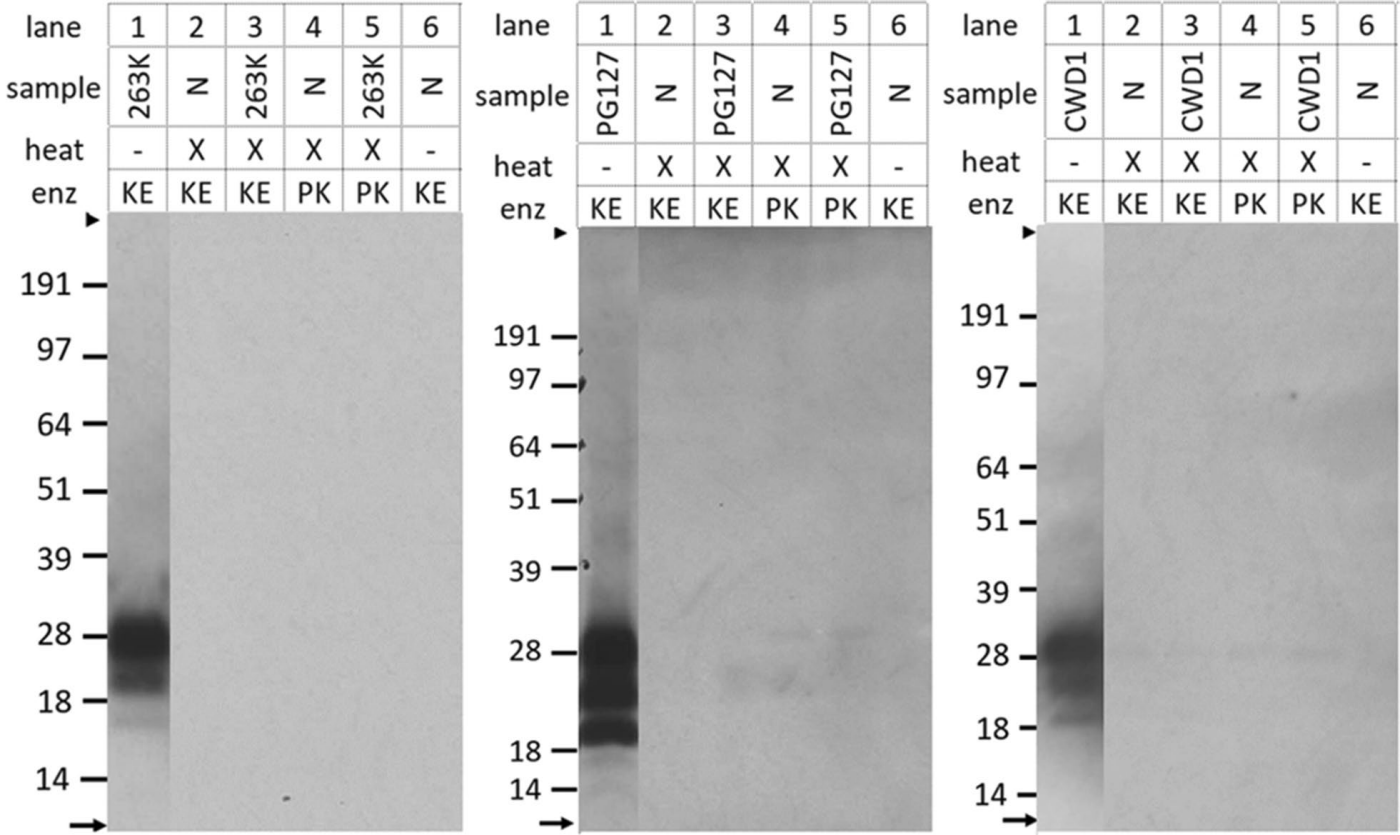
**Proteolytic digestion of PrP**^**Sc**^** in brain homogenates infected with PG127 scrapie, 263 K scrapie and CWD1.** Heated and digested samples are indicated with an X. Three blots from left to right: sheep PG127, hamster 263 K and bank vole CWD1 immunostained with respectively Sha31, a mix of 3F4 and Sha31, and SAF84. Antibody concentrations 0.5 µg mL^−1^, except 3F4 at 1.0 µg mL^−1^. Enz-row shows where proteinase K or keratinase was used for digestion. N in lanes 2, 4, and 6 means TSE negative brain samples. Migration position of molecular mass markers are indicated at the left together with their kDa (SeeBlue markers). Gels were run in MOPS buffer. Tissue equivalents applied: 455 µg mL^−1^. Per panel lanes 1 and 2–6 are from the same blot.

To see whether retention of infectivity also would occur in similarly autoclaved TSEs from other sources we chose rapid infection models which were hamster 263 K scrapie in hamsters, sheep PG127 (or Dawson) scrapie in Tg338 shPrP_VRQ_ mice, and bank vole CWD1_109I_ in 109I/I bank voles (Bv109I) with reported minimal incubation times of less than 100 days. The effect of heat treatment and that of heat treatment plus digestion were checked by Western blotting which confirmed the effectiveness of the PrP^Sc^ removal by KE as well as proteinase K (Figure 5). The ^10^logID_50_ g^−1^ titers of non-heated inocula were for 263 K, PG127 scrapie and CWD1_109I_ respectively 6–6.5, 5.8–6.3 and 8.4 (Table 3). However, heat treatment at 115 °C in each of the three systems led already to an infectivity reduction below the detection limit except maybe for bank voles where one out of six animals was positive at highest concentration tested corresponding to a titer of roughly 3.4 ^10^logID_50_ g^−1^, which in that case would mean an infectivity reduction of at least 5 ^10^log units. Subsequent digestion with KE or PK of all heated inocula yielded TSE negative outcomes for clinical signs and PrP^res^ testing in Western blots.

**Table 3:** Infectivity of heat and protease treated whole brain homogenates in the host rodent species

Infection model	Treatment				
	Detergent, no heat	Detergent, heat	Detergent, heat, KE	Detergent, heat, PK	NEG Cntrls^a^
263 K in hamsters	6/6 (ID_50_ = 6.0–6.5)	0/6 (ID_50_ < 3.5)	0/6 (ID_50_ < 3.5)	0/6 (ID_50_ < 3.5)	0/12 (ID_50_ < 3.5)
	117 ± 7 d	> 325 d	> 325 d	> 325 d	> 284 d
PG127 scrapie in tg338 mice	6/6 (ID_50_ = 5,8–6,3)	0/6 (ID_50_ < 2,5 ID)	0/6 (ID_50_ < 2,5 ID)	0/6 (ID_50_ < 2,5 ID)	0/12 (ID_50_ < 2.5)
	82 ± 4 d	> 200 d	> 200 d	> 200 d	> 200 d
^109I^CWD1 in Bv109I bank voles	6/6^b^ (ID_50_ = 8.4)	1/6 (ID_50_ = 3.4)	0/6 (ID = < 3)	0/6 (ID = < 3)	0/12 (ID_50_ < 3)
	44 ± 4 d	318 d	> 450 d	> 450 d	> 450 d

Inocula tested at 1% tissue concentration, except where indicated. Titres were based on survival times and deduced for hamster 263 K scrapie as in [1], for sheep PG127 scrapie from end-point titration curve in [43], and for CWD1_109I_ from end-point titration curve in [42].

^a^12 animals, three in each of the four treatments.

^b^unheated material tested with 0.1% inoculum; other treatments with bank voles were in addition to 1% also checked at 0.1 and 0.01% tissue concentrations and all with no attacks as result.

These experiments do show that BSE differs from the other three TSEs with respect to resistance of infectivity to heat (i.c. 115 °C for 40 min under wet conditions and in the presence of detergent), a process that allowed proteolytic removal of PrP^Sc^ below the detection limits of the tests used.

## Discussion

In bovine BSE infected brain homogenate heated under wet conditions at 115 °C for 40 min a high level of infectivity was retained when inoculated in transgenic mice (Tgbov XV mice) expressing bovine PrP. This high level of residual heat resistant infectivity was not further inactivated by exhaustive proteolytic removal of PrP^Sc^. In addition, the molecular BSE-strain type of PrP^res^ appeared conserved in the mice. Similarly treated brain from three other prion sources with short incubation times in rodents lost their infectivity by the heat treatment, confirming the unique heat resistance of the BSE agent from cattle compared to that of other prions.

The loss of titre in bovine BSE brain homogenates after heating at 115 °C in the presence 2% sarkosyl amounted to only 0.7–1.8 ^10^log ID_50_ g^−1^. Incomplete BSE inactivation tested in Tgbov XV mice compares well with other studies in homogenates using heating at temperatures between 100–140 °C for either bovine BSE in wild type mice and rodent BSE in transgenic mice expressing high levels of murine PrP [49–51]. The infectivity loss of scrapie types 263 K and PG127, and CWD1_109I_ agrees with studies that BSE carries an exceptional resistance to wet heat conditions compared to many other TSE strains that get largely inactivated already below 100 °C [49, 52–55].

Remarkably, removal of PrP^Sc^ with keratinase after heating at 115 °C from BSE and other TSE strains did confirm the effectivity of the enzymatic digestion with keratinase (and proteinase K) when tested in Western blotting and, in case of BSE, in commercially available diagnostic tests. It may be that in the BSE material after heating and keratinase digestion some form(s) of PrP^Sc^ were preserved and remained undetectable. Protein material was found in both low molecular mass region < 6 kDa and in the > 300 kDa protein fraction, but both were not immunoreactive with PrP-specific antibodies. Also, additional testing for the presence of PrP using three different sensitive biochemical diagnostic ELISA’s did not reveal residual signs of PrP^Sc^. Furthermore, an extra dissociation and unfolding treatment on Western blot PVDF membranes with guanidinium thiocyanate also did not lead to any binding of PrP specific antibody (not shown). If sub-background amounts of PrP^Sc^ or fragments thereof still were present in the inoculum, then the difference between infectivity reduction by heat (3–60 fold decrease) and PrP^Sc^ by subsequent breakdown with keratinase (333–2500 fold decrease) does not well correlate since these differences between the bioassay and biochemical data range between 5–800 fold. This was also observed by other studies with BSE, where infectivity and PrP-immunoreactivity cannot be simply compared in contrast to prion seeding assays like protein misfolding cyclic amplification (PMCA) and infectivity testing [49, 56]^1^*.* If PrP gets fully removed by our method—which is difficult to prove by the relative limited sensitivity of antibodies compared to infectivity testing in transgenic mice—other molecular entities should still be present that attribute to the PrP^C^ to PrP^Sc^ conversion. Nevertheless, the discrepancy between infectivity and PrP^Sc^ reduction in our study and another study [24] is quite large which justifies searching for alternative factors or cofactors that promote PrP^Sc^ propagation depending on strain and environment. In fact, molecules like phospholipid, dextran sulfate and RNA have been reported to be involved in in vitro PrP^Sc^ prion formation and infectivity [4, 9, 18, 20, 49]. In this respect BSE represents a unique example of a prion agent that it is able to transmit to many other species and to induce PrP-PrP^Sc^ conversion within one species irrespective PrP-polymorphisms [57, 58].

The bovine BSE-typical molecular PrP triple band profile and molecular masses were retained in the brains of transgenic mice expressing bovine PrP when inoculated with heat and keratinase treated bovine BSE brain material. Also, the clinical signs observed were similar in the different inocula used. This corroborates observations about thermostability of strains and the hypothesis that the prion agent contains both a host (i.e. PrP) and a strain dependent component, the latter of which could be a non-protein component [24, 54].

Infectivity was not tested in KE-digested BSE samples that were not heated since we were focused on preparations where PrP was absent as an opportunity to produce a process for significant removal of BSE infectivity. Such test could have yielded information whether in case of BSE a proteolytic digestion of non-heated prion material had lead to infectivity reduction as is the case for e.g. with purified hamster scrapie material [5]. Nevertheless, we did test for the presence of PrP^Sc^ in such samples by Western blots where the level of PrP-reactive material did hardly differ between non-heated digested and heated non-digested or partly-digested material, which suggests that infectivity also would have been retained in the non-heated material after digestion.

An approximately tenfold titre increase of infectivity was observed after keratinase treatment of the heated BSE samples which appeared reproducible in a second experiment (from 10^4.6^ to 10^5.7^ ID_50_ g^−1^, Table 2 and Figure 3). This increase leads us to some assumptions. Possibly there was still an undetectable amount of PrP^Sc^ present that under the conditions of the proteolytic treatment attained an increased PrP seeding capacity. Another more complicated possibility would be that after heating a strain determining factor for prion formation was released in the brain homogenate by the protease used. In that case, new PrP^Sc^ could be generated from PrP in the transgenic mouse brain leading to de novo infectivity while BSE strain properties remained the same. This latter situation also requires a complete conversion process leading to an infectivity titre higher than before proteolysis. The factor most probably is a macromolecular product still present in the pellet after methanol treatment.

The protein only theory has allowed to better understand the nature and origin of prion agents with PrP^Sc^ as the carrier of infectivity. As example of the validity of the prion hypothesis is the application of the one gene—one protein concept in the successful Mendelian way of breeding for resistance towards the disappearance of scrapie in sheep and observation of increased levels of a resistance related polymorphism in humans in the epidemic kuru region in Papua New Guinea [15, 16]. Yet, explaining the molecular basis of strains with their phenotypical behavior based on a polymorphic appearance of PrP^Sc^ remains a challenge. Here, the exceptional resistance of BSE to heat and the subsequent removal of PrP^Sc^ makes this prion type a rather unique substrate for solving these prion strain questions^1^. The answers could be found in the precipitable leftover material of the heated and digested BSE infected whole brain preparations. Thus, varying heat treatment and the enzymatic digestion conditions such as pH, detergent and choice of protease could well be of use in figuring out which additional molecular fraction can modulate the PrP^C^ to PrP^Sc^ conversion towards strain related properties. Maybe other heat resistant strains than BSE could serve this aim with the potential practical advantage of absence of zoonotic behavior.

## Supplementary Information

**Additional file 1. Principle of the three ELISA tests to detect bovine PrP**^**Sc**^** and PrP**^**res**^**.**

**Additional file 2. PrP**^**Sc**^** digestion by keratinase of bovine brain after heat treatment at 115 °C.** BSE infected brain in lanes 4, 7 and 10-13, negative control brain in lanes 2 and 3. Material in lanes 2 and 4 was heated at 115 °C in presence of detergent before digestion. Lanes 3 and 10-13: non-heated material digested by KE. In the heated BSE sample in lane 4 no PrP-specific immunoreactivity has remained neither throughout the lane nor in the high molecular mass region at the top, while in the non-heated material there was (lane 10). Lanes 1 and 9, mixture of molecular mass markers SeeBlue and MagicMark XP for which migration positions are indicated in kDa at the left; lanes 5, 6 and 8, no sample applied. Top and running front are indicated with arrow heads and arrows, respectively. Antibody used: 94B4 (0.2 μg mL^-1^). Gel was run in MOPS buffer. Symbols: TE = tissue equivalents; MM = molecular mass markers; KE = keratinase; + = treatment applied.

**Additional file 3. Survival times of diseases Tgbov XV mice in the treatment groups: no detergent (positive control), detergent plus heat, and detergent plus keratinase.**

## Footnotes

1 When in revision, a paper appeared where no PrP^Sc^ could be found in one out of three clinically positive steers orally infected with bovine BSE. In this one animal only by PMCA a weak positive signal for PrP^Sc^ was found in the 3rd round at highest seeding concentration while Tg BovXV mice remained negative for this steer. The two other steers were clearly PrP^Sc^ positive in biochemical tests, immunohistochemistry, PMCA at 1^st^, 2^nd^ and 3^rd^ round, and were infective in the bovinized mice [59].

## References

[1] Prusiner SB (1982). Novel proteinaceous infectious particles cause scrapie. Science.

[2] Brown P, Liberski PP, Wolff A, Gajdusek DC (1990). Conservation of infectivity in purified fibrillary extracts of scrapie-infected hamster brain after sequential enzymatic digestion or polyacrylamide gel electrophoresis. Proc Natl Acad Sci USA.

[3] Griffith JS (1967). Self-replication and scrapie. Nature.

[4] Ma J, Wang F (2014). Prion disease and the 'protein-only hypothesis'. Essays Biochem.

[5] McKinley MP, Bolton DC, Prusiner SB (1983). A protease-resistant protein is a structural component of the scrapie prion. Cell.

[6] Avar M, Heinzer D, Steinke N, Dogancay B, Moos R, Lugan S, Cosenza C, Hornemann S, Andreoletti O, Aguzzi A (2020). Prion infection, transmission, and cytopathology modeled in a low-biohazard human cell line. Life Sci Alliance.

[7] Bueler H, Aguzzi A, Sailer A, Greiner RA, Autenried P, Aguet M, Weissmann C (1993). Mice devoid of PrP are resistant to scrapie. Cell.

[8] Deleault NR, Harris BT, Rees JR, Supattapone S (2007). Formation of native prions from minimal components in vitro. Proc Natl Acad Sci U S A.

[9] Fernandez-Borges N, Di Bari MA, Erana H, Sanchez-Martin M, Pirisinu L, Parra B, Elezgarai SR, Vanni I, Lopez-Moreno R, Vaccari G, Venegas V, Charco JM, Gil D, Harrathi C, D’Agostino C, Agrimi U, Mayoral T, Requena JR, Nonno R, Castilla J (2018). Cofactors influence the biological properties of infectious recombinant prions. Acta Neuropathol.

[10] Kim JI, Cali I, Surewicz K, Kong Q, Raymond GJ, Atarashi R, Race B, Qing L, Gambetti P, Caughey B, Surewicz WK (2010). Mammalian prions generated from bacterially expressed prion protein in the absence of any mammalian cofactors. J Biol Chem.

[11] Makarava N, Kovacs GG, Bocharova O, Savtchenko R, Alexeeva I, Budka H, Rohwer RG, Baskakov IV (2010). Recombinant prion protein induces a new transmissible prion disease in wild-type animals. Acta Neuropathol.

[12] Salvesen O, Espenes A, Reiten MR, Vuong TT, Malachin G, Tran L, Andreoletti O, Olsaker I, Benestad SL, Tranulis MA, Ersdal C (2020). Goats naturally devoid of PrP^C^ are resistant to scrapie. Vet Res.

[13] Wang F, Wang X, Yuan CG, Ma J (2020). Generating a prion with bacterially expressed recombinant prion protein. Science.

[14] Watts JC, Giles K, Stohr J, Oehler A, Bhardwaj S, Grillo SK, Patel S, DeArmond SJ, Prusiner SB (2012). Spontaneous generation of rapidly transmissible prions in transgenic mice expressing wild-type bank vole prion protein. Proc Natl Acad Sci U S A.

[15] Hagenaars TJ, Melchior MB, Windig JJ, Bossers A, Davidse A, van Zijderveld FG (2018). Modelling of strategies for genetic control of scrapie in sheep: the importance of population structure. PLoS ONE.

[16] Mead S, Whitfield J, Poulter M, Shah P, Uphill J, Campbell T, Al-Dujaily H, Hummerich H, Beck J, Mein CA, Verzilli C, Whittaker J, Alpers MP, Collinge J (2009). A novel protective prion protein variant that colocalizes with kuru exposure. N Engl J Med.

[17] Ricci A, Allende A, Bolton D, Chemaly M, Davies R, Salvador Fernandez Escamez P, Girones R, Herman L, Koutsoumanis K, Lindqvist R, Nørrung B, Robertson L, Ru G, Sanaa M, Skandamis P, Speybroeck N, Simmons M, Ter Kuile B, Threlfall J, Wahlstrom H, Acutis P-L, Andreoletti O, Goldmann W, Langeveld J, Windig JJ, Ortiz Pelaez A, Snary E (2017). Genetic resistance to transmissible spongiform encephalopathies (TSE) in goats. EFSA J.

[18] Deleault NR, Walsh DJ, Piro JR, Wang F, Wang X, Ma J, Rees JR, Supattapone S (2012). Cofactor molecules maintain infectious conformation and restrict strain properties in purified prions. Proc Natl Acad Sci U S A.

[19] Hunter GD, Gibbons RA, Kimberlin RH, Millson GC (1969). Further studies of the infectivity and stability of extracts and homogenates derived from scrapie affected mouse brains. J Comp Pathol.

[20] Simoneau S, Thomzig A, Ruchoux MM, Vignier N, Daus ML, Poleggi A, Lebon P, Freire S, Durand V, Graziano S, Galeno R, Cardone F, Comoy E, Pocchiari M, Beekes M, Deslys JP, Fournier JG (2015). Synthetic scrapie infectivity: interaction between recombinant PrP and scrapie brain-derived RNA. Virulence.

[21] Lasmezas CI, Deslys JP, Robain O, Jaegly A, Beringue V, Peyrin J, Fournier JG, Hauw JJ, Rossier J, Dormont D (1997). Transmission of the BSE agent to mice in the absence of detectable abnormal prion protein. Science.

[22] Langeveld JP, Wang JJ, Van de Wiel DF, Shih GC, Garssen GJ, Bossers A, Shih JC (2003). Enzymatic degradation of prion protein in brain stem from infected cattle and sheep. J Infect Dis.

[23] Dickinson J, Murdoch H, Dennis MJ, Hall GA, Bott R, Crabb WD, Penet C, Sutton JM, Raven ND (2009). Decontamination of prion protein (BSE301V) using a genetically engineered protease. J Hosp Infect.

[24] Miyazawa K, Emmerling K, Manuelidis L (2011). High CJD infectivity remains after prion protein is destroyed. J Cell Biochem.

[25] Sklaviadis TK, Manuelidis L, Manuelidis EE (1989). Physical properties of the Creutzfeldt-Jakob disease agent. J Virol.

[26] Silveira JR, Raymond GJ, Hughson AG, Race RE, Sim VL, Hayes SF, Caughey B (2005). The most infectious prion protein particles. Nature.

[27] Colby DW, Prusiner SB (2011) Prions. In: Morimoto R, Kelly J, Selkoe D (eds), Cold Spring Harb Perspect Biol pp 1–23 10.1101/cshperspect.a006833 PMC3003464 21421910

[28] Cronier S, Gros N, Tattum MH, Jackson GS, Clarke AR, Collinge J, Wadsworth JD (2008). Detection and characterization of proteinase K-sensitive disease-related prion protein with thermolysin. Biochem J.

[29] Leske H, Hornemann S, Herrmann US, Zhu C, Dametto P, Li B, Laferriere F, Polymenidou M, Pelczar P, Reimann RR, Schwarz P, Rushing EJ, Wuthrich K, Aguzzi A (2017). Protease resistance of infectious prions is suppressed by removal of a single atom in the cellular prion protein. PLoS ONE.

[30] Demart S, Fournier JG, Creminon C, Frobert Y, Lamoury F, Marce D, Lasmezas C, Dormont D, Grassi J, Deslys JP (1999). New insight into abnormal prion protein using monoclonal antibodies. Biochem Biophys Res Commun.

[31] Feraudet C, Morel N, Simon S, Volland H, Frobert Y, Creminon C, Vilette D, Lehmann S, Grassi J (2005). Screening of 145 anti-PrP monoclonal antibodies for their capacity to inhibit PrPSc replication in infected cells. J Biol Chem.

[32] Harmeyer S, Pfaff E, Groschup MH (1989). Synthetic peptide vaccines yield monoclonal antibodies to cellular and pathological prion proteins of ruminants. J Gen Virol.

[33] Jacobs JG, Bossers A, Rezaei H, van Keulen LJ, McCutcheon S, Sklaviadis T, Lantier I, Berthon P, Lantier F, van Zijderveld FG, Langeveld JP (2011). Proteinase K-resistant material in ARR/VRQ sheep brain affected with classical scrapie is composed mainly of VRQ prion protein. J Virol.

[34] Jacobs JG, Langeveld JP, Biacabe AG, Acutis PL, Polak MP, Gavier-Widen D, Buschmann A, Caramelli M, Casalone C, Mazza M, Groschup M, Erkens JH, Davidse A, van Zijderveld FG, Baron T (2007). Molecular discrimination of atypical bovine spongiform encephalopathy strains from a geographical region spanning a wide area in Europe. J Clin Microbiol.

[35] Kascsak RJ, Rubenstein R, Merz PA, Tonna-DeMasi M, Fersko R, Carp RI, Wisniewski HM, Diringer H (1987). Mouse polyclonal and monoclonal antibody to scrapie-associated fibril proteins. J Virol.

[36] O’Rourke KI, Baszler TV, Besser TE, Miller JM, Cutlip RC, Wells GA, Ryder SJ, Parish SM, Hamir AN, Cockett NE, Jenny A, Knowles DP (2000). Preclinical diagnosis of scrapie by immunohistochemistry of third eyelid lymphoid tissue. J Clin Microbiol.

[37] Thuring CM, van Keulen LJ, Langeveld JP, Vromans ME, van Zijderveld FG, Sweeney T (2005). Immunohistochemical distinction between preclinical bovine spongiform encephalopathy and scrapie infection in sheep. J Comp Pathol.

[38] Slootstra JW, Puijk WC, Ligtvoet GJ, Langeveld JP, Meloen RH (1996). Structural aspects of antibody-antigen interaction revealed through small random peptide libraries. Mol Divers.

[39] Lin X, Lee CG, Casale ES, Shih JC (1992). Purification and characterization of a keratinase from a feather-degrading *Bacillus licheniformis* strain. Appl Environ Microbiol.

[40] Wang JJ, Shih J (1999). Fermentation production of keratinase from *Bacillus licheniformis* PWD-1 and a recombinant *B. subtilis* FDB-29. J Ind Microbiol Biotechnol.

[41] Buschmann A, Groschup MH (2005). Highly bovine spongiform encephalopathy-sensitive transgenic mice confirm the essential restriction of infectivity to the nervous system in clinically diseased cattle. J Infect Dis.

[42] Hubert JJ (1992). Bioassay.

[43] Andreoletti O, Orge L, Benestad SL, Beringue V, Litaise C, Simon S, Le Dur A, Laude H, Simmons H, Lugan S, Corbiere F, Costes P, Morel N, Schelcher F, Lacroux C (2011). Atypical/Nor98 scrapie infectivity in sheep peripheral tissues. PLoS Pathog.

[44] Beekes M, Baldauf E, Diringer H (1996). Sequential appearance and accumulation of pathognomonic markers in the central nervous system of hamsters orally infected with scrapie. J Gen Virol.

[45] Di Bari MA, Nonno R, Castilla J, D’Agostino C, Pirisinu L, Riccardi G, Conte M, Richt J, Kunkle R, Langeveld J, Vaccari G, Agrimi U (2013). Chronic wasting disease in bank voles: characterisation of the shortest incubation time model for prion diseases. PLoS Pathog.

[46] Laemmli UK (1970). Cleavage of structural proteins during the assembly of the head of bacteriophage T4. Nature.

[47] Switzer RC, Merril CR, Shifrin S (1979). A highly sensitive silver stain for detecting proteins and peptides in polyacrylamide gels. Anal Biochem.

[48] Thomzig A, Kratzel C, Lenz G, Kruger D, Beekes M (2003). Widespread PrPSc accumulation in muscles of hamsters orally infected with scrapie. EMBO Rep.

[49] Marin-Moreno A, Aguilar-Calvo P, Moudjou M, Espinosa JC, Beringue V, Torres JM (2019). Thermostability as a highly dependent prion strain feature. Sci Rep.

[50] Schreuder BE, Geertsma RE, van Keulen LJ, van Asten JA, Enthoven P, Oberthur RC, de Koeijer AA, Osterhaus AD (1998). Studies on the efficacy of hyperbaric rendering procedures in inactivating bovine spongiform encephalopathy (BSE) and scrapie agents. Vet Rec.

[51] Taylor DM, Fraser H, McConnell I, Brown DA, Brown KL, Lamza KA, Smith GR (1994). Decontamination studies with the agents of bovine spongiform encephalopathy and scrapie. Arch Virol.

[52] Fernie K, Steele PJ, Taylor DM, Somerville RA (2007). Comparative studies on the thermostability of five strains of transmissible-spongiform-encephalopathy agent. Biotechnol Appl Biochem.

[53] Matsuura Y, Ishikawa Y, Murayama Y, Yokoyama T, Somerville RA, Kitamoto T, Mohri S (2020). Eliminating transmissibility of bovine spongiform encephalopathy by dry-heat treatment. J Gen Virol.

[54] Somerville RA, Gentles N (2011). Characterization of the effect of heat on agent strains of the transmissible spongiform encephalopathies. J Gen Virol.

[55] Taylor DM, Fernie K, Steele PJ, McConnell I, Somerville RA (2002). Thermostability of mouse-passaged BSE and scrapie is independent of host PrP genotype: implications for the nature of the causal agents. J Gen Virol.

[56] Ackermann I, Shawulu JC, Keller M, Fatola OI, Groschup MH, Balkema-Buschmann A (2018) Exploring PMCA as a potential in-vitro alternative method to mouse bioassays for the highly sensitive detection of BSE prions. Berl Münch Tierärztl Wochensch O/A-131:9/10, DOI 10.2376/0005-9366-18021

[57] Priem J, Langeveld JP, van Keulen LJ, van Zijderveld FG, Andreoletti O, Bossers A (2014). Enhanced virulence of sheep-passaged bovine spongiform encephalopathy agent is revealed by decreased polymorphism barriers in prion protein conversion studies. J Virol.

[58] Torres JM, Espinosa JC, Aguilar-Calvo P, Herva ME, Relano-Gines A, Villa-Diaz A, Morales M, Parra B, Alamillo E, Brun A, Castilla J, Molina S, Hawkins SA, Andreoletti O (2014). Elements modulating the prion species barrier and its passage consequences. PLoS ONE.

[59] Dudas S, Anderson R, Staskevicus A, Mitchell G, Cross JC, Czub S (2021). Exploration of genetic factors resulting in abnormal disease in cattle experimentally challenged with bovine spongiform encephalopathy. Prion.

