# Supplementary material for: Stability of BSE infectivity towards heat treatment even after proteolytic removal of prion protein
Source: Vet Res. 2021 Apr 16;52:59. doi: 10.1186/s13567-021-00928-8 (PMC8052740; doi:10.1186/s13567-021-00928-8)
Supplement: Supplementary file 1 — Additional file 1. Principle of the three ELISA tests to detect bovine PrPSc and PrPres. [file 13567_2021_928_MOESM1_ESM.docx]

**Additional file 1 Principle of the three ELISA tests to detect bovine PrP^Sc^ and PrP^res^**

The term PrP^res^ is used when in the commercial test a digestion step with proteinase K is included to effectively breakdown PrP^C^. The removal of the N‑terminal region of (the PrP units in) PrP^Sc^ is then a consequence, such that epitopes for e.g. SAF34, P4 and 12B2 are variably absent depending on the stringency of the digestion conditions. Epitopes in the remaining C‑terminal part remain intact. When a digestion with proteinase K under stringent conditions is performed, N-terminal epitopes are removed for e.g. SAF34 (62QPHGGGW92) and 12B2 (101WGQGG105), but not for 9A2 (110WNK112) and other antibodies with specificity to the more C-terminal region of PrP downstream the 9A2 epitope.

The TeSeE test will detect presence of PrP^res^ after subsequent steps of immobilisation by precoated antibody SAF34, denaturation, peroxidase-conjugated antibody Bar224 binding, incubation with substrate and spectrophotometry. Digestion with proteinase K is an elementary step which is performed under less stringent digestion conditions.

The HerdCheck test captures fibrillar proteins such as PrP^Sc^ and PrP^res^ in prions using 96 wells titre plates with immobilised Seprion ligand on the wall. After binding of prion material to the ligand a protein denaturation treatment follows to allow access to PrP-specific monoclonal antibodies, incubation with peroxidase conjugated anti-mouse IgG, substrate addition and final spectrophotometric detection. The specificity of the PrP specific antibody(ies) used is (are) not disclosed. No proteinase K treatment is applied.

The CediTect test measures unfolding of PrP^res^ aggregates after adsorption to 96 well polyvinylidenefluoride filter‑plates (PVDF) that can be washed by filtration for subsequent steps. Two wells with the same sample are differently treated to establish a measure for protein unfolding by comparing non‑denatured (n) using PBS and denatured (d) state using 4 M guanidine-thiocyanate. Further subsequent steps with intermediate washing are incubations respectively with primary antibody (94B4 or 9A2), rabbit anti‑mouse IgG conjugated to alkaline phosphatase, and Luminol. Chemoluminescence detection was carried out with a Centro LB 960 Luminometer (Berthold, Germany). After denaturation PrP^res^ epitopes become highly accessible for PrP‑specific primary antibodies yielding high d/n ratios in BSE positive cases.
