# Supplementary material for: Stability of BSE infectivity towards heat treatment even after proteolytic removal of prion protein
Source: Vet Res. 2021 Apr 16;52:59. doi: 10.1186/s13567-021-00928-8 (PMC8052740; doi:10.1186/s13567-021-00928-8)
Supplement: Supplementary file 2 — Additional file 2. PrPSc digestion by keratinase of bovine brain after heat treatment at 115 °C. BSE infected brain in lanes 4, 7 and 10-13, negative control brain in lanes 2 and 3. Material in lanes 2 and 4 was heated at 115 °C in presence of detergent before digestion. Lanes 3 and 10-13: non-heated material digested by KE. In the heated BSE sample in lane 4 no PrP-specific immunoreactivity has remained neither throughout the lane nor in the high molecular mass region at the top, while in the non-heated material there was (lane 10). Lanes 1 and 9, mixture of molecular mass markers SeeBlue and MagicMark XP for which migration positions are indicated in kDa at the left; lanes 5, 6 and 8, no sample applied. Top and running front are indicated with arrow heads and arrows, respectively. Antibody used: 94B4 (0.2 μg mL-1). Gel was run in MOPS buffer. Symbols: TE = tissue equivalents; MM = molecular mass markers; KE = keratinase; + = treatment applied. [file 13567_2021_928_MOESM2_ESM.docx]

Additional file 2: PrPSc digestion by keratinase of bovine brain after heat treatment at 115 oC. BSE infected brain in lanes 4, 7 and 10-13, negative control brain in lanes 2 and 3. Material in lanes 2 and 4 was heated at 115 oC in presence of detergent before digestion. Lanes 3 and 10-13: non-heated material digested by KE. In the heated BSE sample in lane 4 no PrP-specific immunoreactivity has remained neither throughout the lane nor in the high molecular mass region at the top, while in the non-heated material there was (lane 10). Lanes 1 and 9, mixture of molecular mass markers SeeBlue and MagicMark XP for which migration positions are indicated in kDa at the left; lanes 5, 6 and 8, no sample applied. Top and running front are indicated with arrow heads and arrows, respectively. Antibody used: 94B4 (0.2 μg mL-1). Gel was run in MOPS buffer. Symbols: TE = tissue equivalents; MM = molecular mass markers; KE = keratinase; + = treatment applied.
