# Supplementary material for: Stability of BSE infectivity towards heat treatment even after proteolytic removal of prion protein
Source: Vet Res. 2021 Apr 16;52:59. doi: 10.1186/s13567-021-00928-8 (PMC8052740; doi:10.1186/s13567-021-00928-8)
Supplement: Supplementary file 3 — Additional file 3. Survival times of diseases Tgbov XV mice in the treatment groups: no detergent (positive control), detergent plus heat, and detergent plus keratinase. [file 13567_2021_928_MOESM3_ESM.docx]

**Additional file 3 Survival times of diseases Tgbov XV mice in the treatment groups: no detergent (positive control), detergent plus heat, and detergent plus keratinase^a^.**

^a^ Values of survival time are in days and given as average ± SD with between parentheses the range. Infection rate (IR) reflects the total number of positives per total animals in the dose group. Dose is expressed as ^10^log dilution factor of wet brain tissue. Inoculated negative controls survived 622 ± 105 days (dose -2; survival range 366-725 d; *n* = 10).
